# Supplementary material for: An integrated workflow for crosslinking mass spectrometry
Source: Mol Syst Biol. 2019 Sep 20;15(9):e8994. doi: 10.15252/msb.20198994 (PMC6753376; doi:10.15252/msb.20198994)
Supplement: Supplementary file 1 — Appendix [file MSB-15-e8994-s001.pdf]

Appendix

An integrated workflow for crosslinking mass spectrometry

Marta L. Mendes[1,a,#], Lutz Fischer[1,2,#], Zhuo A. Chen[1], Marta Barbon[3,4], Francis J. O'Reilly[1], Sven Giese[1], Michael Bohlke-Schneider[1], Adam Belsom[1,2], Therese Dau[2], Colin W. Combe[2], Martin Graham[2], Markus R. Eisele[5], Wolfgang Baumeister[5], Christian Speck[3,4], Juri Rappsilber[1,2,*]

[1] Bioanalytics, Institute of Biotechnology, Technische Universität Berlin, 13355 Berlin, Germany

[2] Wellcome Centre for Cell Biology, University of Edinburgh, Edinburgh EH9 3BF, United Kingdom

[3] MRC London Institute of Medical Sciences (LMS), London, UK

[4] DNA Replication Group, Institute of Clinical Sciences (ICS), Faculty of Medicine, Imperial College London, London, UK

[5] Department of Molecular Structural Biology, Max Planck Institute of Biochemistry, 82152 Martinsried, Germany

[a] Current address: Proteome and Genome Research Unit, Luxembourg Institute of Health, Luxembourg, Luxembourg

[#] Marta L. Mendes and Lutz Fischer contributed equally to this work

* Corresponding author: juri.rappsilber@tu-berlin.de

Table of contents

Appendix Table S1 2

Appendix Table S2 5

Appendix Figure S1 6

Appendix Figure S2 7

Appendix Figure S3 9

Appendix Figure S4 10

Appendix Figure S5 11

Appendix Figure S6 11

Step	Description	Note
Pre-requirements	JDK 1.8 or 1.9	Download from:<br>https://www.java.com/ note that when downloading with a 32-bit browser, an explicit selection of a 64-bit download is required.<br>Ensure that java defaults to the correct version. Check version e.g. by running:<br>java -version<br>in the terminal or cmd.
	Enough system memory	For large scale analysis a computer with 16 GB memory is recommended.
Download xiSEARCH	https://rappsilberlab.org/downloads/	
Download xiFDR	https://rappsilberlab.org/downloads/	
Extract	xiSEARCH and xiFDR need to be extracted.<br>No installation is required.	
Start xiSEARCH	Windows: start the supplied 'startXiWindows.bat'	In case the default version is wrong (e.g. <1.8) you can change the path where to find java in the start-script.<br>The start-scripts can be adjusted to define the maximum-heap memory xiSEARCH can use (e.g. –Xmx8G)
	MacOS/Linux open terminal and navigate to folder and start<br>sh startXiUnix.sh	

Appendix Table S1: xiSEARCH Installation

Step	Screenshot	Notes
Start xiSEARCH		If you are using Windows, start the startXiWindows file. If you are using Unix, start the startXiUnix file. This will start Xi with a simple graphical user interface (GUI), and the actual java library (xiSEARCH.jar).
Select output-file	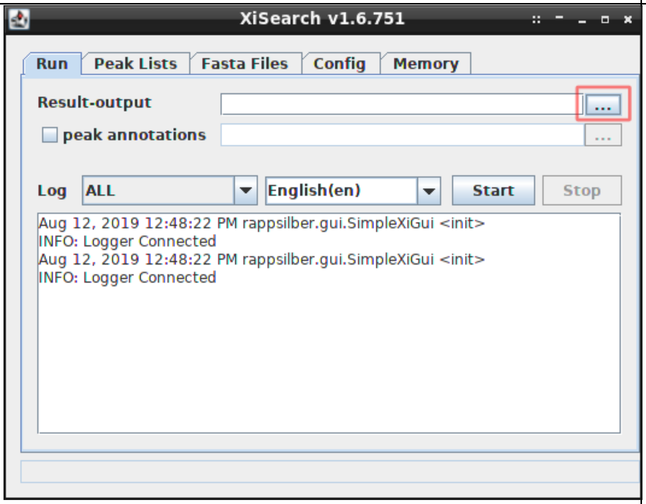	First tab.<br><br>Xi can optionally also output the annotation for spectra. For this tick "peak annotations" and select the output file.
Select peak list	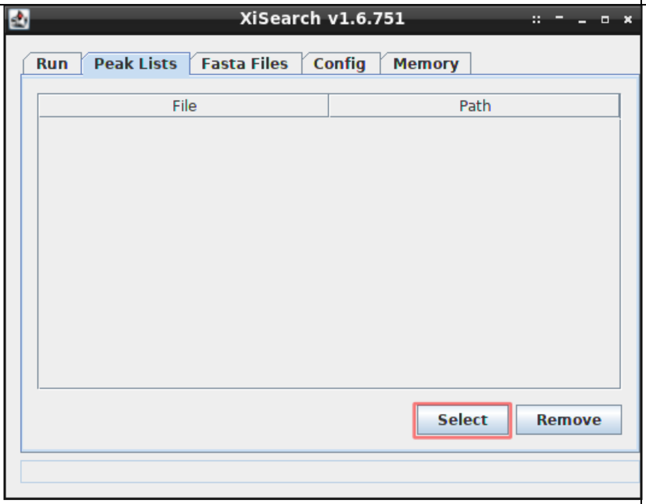	Second Tab<br><br>Xi supports .mgf and MaxQuant .apl files. Further formats are in work.<br><br>The peaklist can be .zip or .gzip compressed.

<table>
<tr><td>Select the FASTA-file</td><td>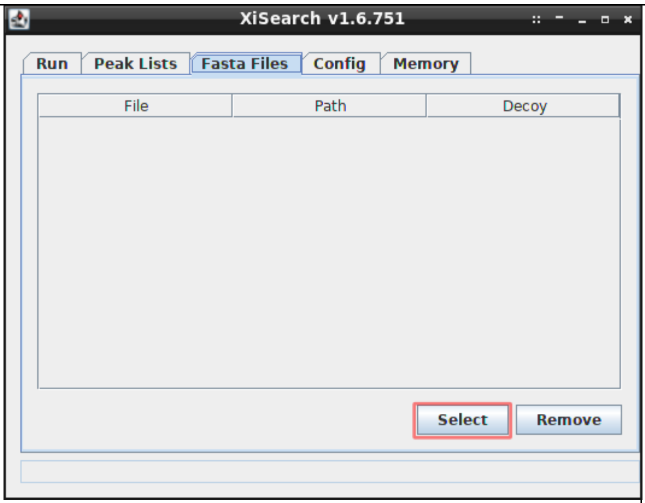
</td><td>Third Tab

Fasta files can be .gzip compressed</td></tr>
<tr><td>Adapt config</td><td>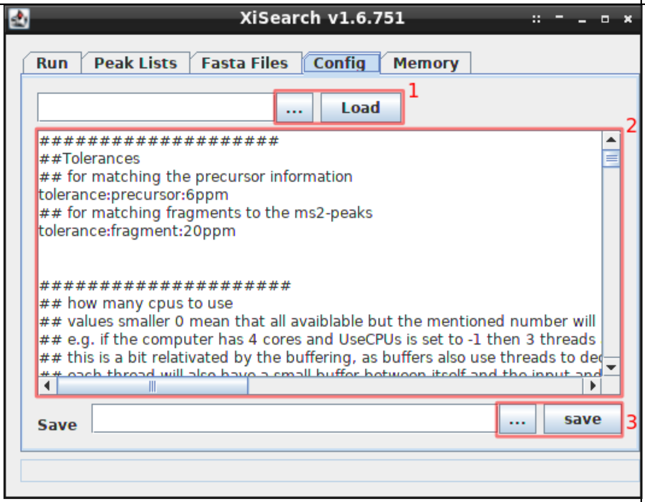
</td><td>You can either load a preexisting config (1) or adapt the default config (2). You should always save (3) the used config together with the search result.

For adapting the default config the most important settings are crosslinker, tolerances and modifications.

Default config defines a search with BS3 as crosslinker, tryptic digest, fixed modification of carbamidomethyl on C and variable modifications of oxidation on M, hydrolysed and amidated BS3 on K, S, T and Y. Ions searched are b and y ions. When using MGF files potentially the two settings

SCAN_RE:

RUN_RE:

need to be adjusted to tell Xi how to find run and scan number</td></tr>
</table>

Start search	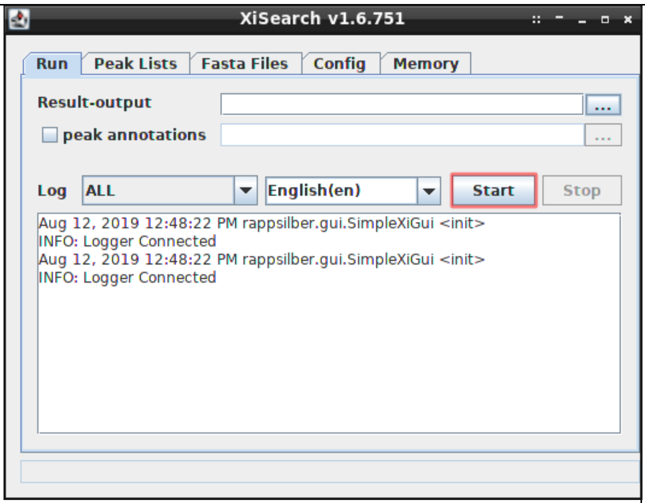	First Tab,<br>On first tab (Run) press "Start"
Wait for completion	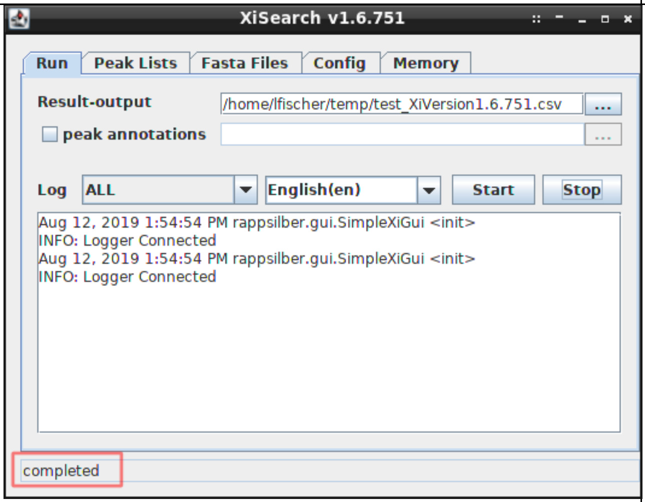	Once the search is completed, the status bar should display "completed" and the "Search" button is enabled again.
xiFDR	xiSEARCH does not assess the FDR. This is done by a separate application, xiFDR (Fischer & Rappsilber, 2017). xiSEARCH result files can be directly read into xiFDR.	
General Notes	• Depending on the size of the sequence-database and the number of search threads, the start-script might need to be adapted to permit Xi to use a larger amount of memory (-Xmx option). This should not exceed the amount of free memory available without running Xi. For example, if a computer has 8 GB of RAM, but per default 5 are used by other programs, then Xi should get at most 3 GB. Otherwise part of the program is likely to be swapped out to disk and in effect will run extremely slow.<br>• Additionally to the GUI, a command line interface is provided that can be used via "java –Xmx4g -jar xiSEARCH.jar --config=[config-file] --peaks=[path to peaklist] --fasta=[path to fasta file] --conf=[some xi-parameters] --output=[csv-file] --peaksout=[csv-file] --exampleconfig=[path]". A description of the arguments can be gained by running java -jar xiSEARCH.jar --help	

Appendix Table S2: xiSEARCH Usage

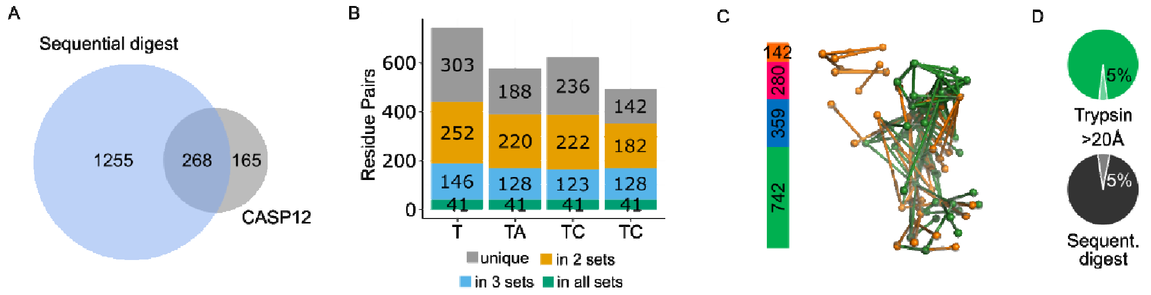

Appendix Figure S1:

High-density CLMS applied to CASP12 target UGGT (PDB|5NV4), using sequential digestion and Xi.

A. Comparison between sequential digestion and CASP12 results. Sequential digestion provides a higher number of unique residue pairs.

B. Overlap of the number of residue pairs for the four digestion conditions in the sequential digestion strategy. Sequential digestion complements trypsin digestion increasing the number of identified unique residue pairs.

C. Sequential digestion workflow applied to high-density CLMS shows a gain by a factor of almost two by adding AspN (blue), chymotrypsin (pink) and GluC (orange) to trypsin (green). Additional residue pairs identified upon sequential digestion (in orange) cover regions of the protein structure not observed with trypsin (in green).

D. The percentage of long-distance residue pairs (> 20 Å) is 5% for both trypsin and sequential digestion, in agreement with our calculated FDR.

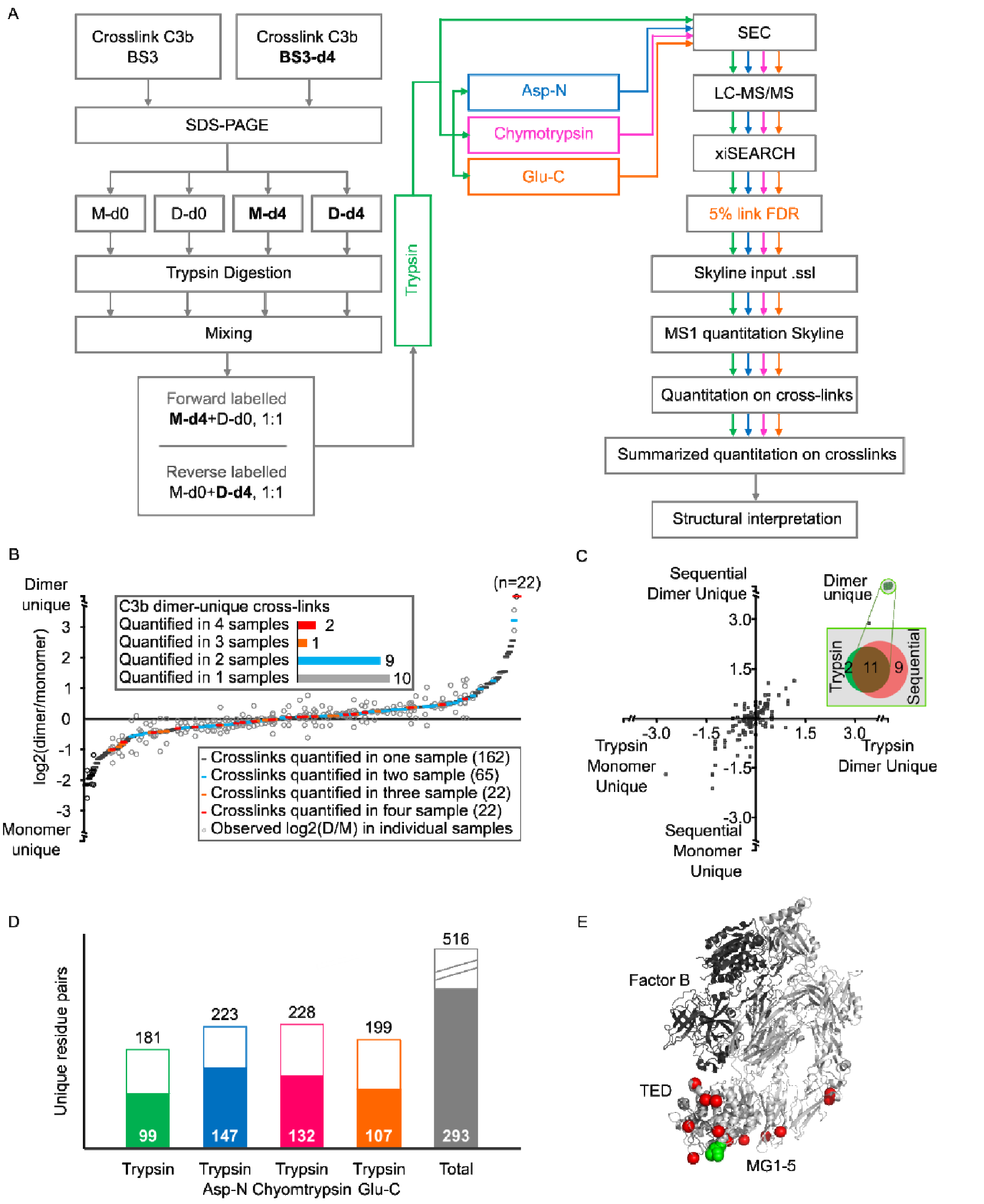

Appendix Figure S2: Sequential digestion applied to QCLMS of C3b dimerization

A. QCLMS-sequential digestion strategy. A C3b sample was divided into two aliquots and crosslinked with either bis[sulfosuccinimidyl] suberate (BS^3) or its deuterated analogue bis[sulfosuccinimidyl] 2,2,7,7-suberate-d4 (BS^3-d4). BS3 and BS3-d4 crosslinked C3b samples were then separately subjected to SDS-PAGE and the monomer and dimer bands of BS3 and BS3-d4 crosslinked C3b were excised and in-gel digested. Based on C3b

abundance, BS3 crosslinked C3b monomer and BS3-d4 crosslinker dimer samples were 1:1 mixed (named as forward-labelled). BS3-d4 crosslinked C3b monomer and BS3 crosslinker dimer samples were also mixed in 1:1 ratio as a reserve-labelled replica. Each of them was divided into four aliquots from which three were further digested each with a second protease. All digested samples were fractioned by SEC chromatography and analysed by LC-MS/MS. Searches were performed by Xi and confidence of identified residue pairs assessed by xiFDR. Quantitation was performed using Skyline.

B. Quantitation reproducibility. The use of different proteases did not lead to major changes on quantified dimer to monomer signal ratios.

C. Comparisons of ratios observed in different sequential digests compared to Trypsin only digest. Green highlighted are links detected as dimer specific.

D. Half of the identified residue pairs per fraction were quantified.

E. C3b (light grey) bound to factor B (dark grey) (PDB|2XWJ) with position of thioester (green) and position of C3b dimer-specific links (red).

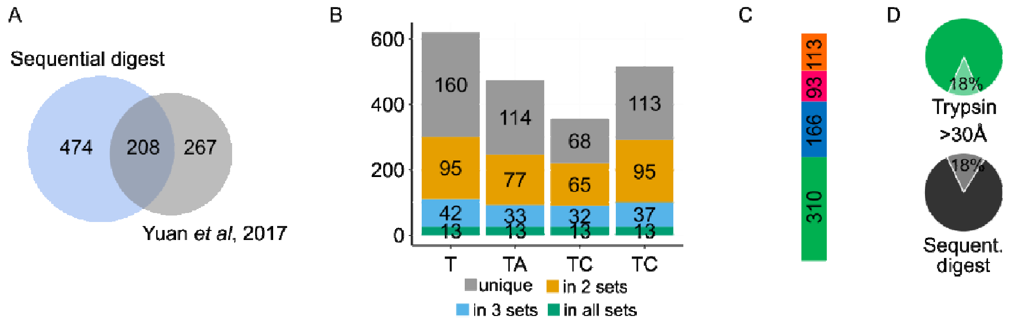

Appendix Figure S3: The OCCM complex analysed by sequential digestion and Xi

A. Comparison between sequential digestion workflow and literature results. Sequential digestion provides a higher number of unique residue pairs.

B. Overlap of the number of residue pairs for the four digestion conditions in the sequential digestion strategy. Sequential digestion complements trypsin digestion increasing the number of identified unique residue pairs.

C. Gain of unique residue pairs by using sequential digestion. The bar represents the number of unique residue pairs gained by adding AspN (blue), chymotrypsin (pink) and GluC (orange) to the digestion with trypsin (green).

D. The percentage of long-distance residue pairs (> 30 Å) is the same for both trypsin and sequential digested samples (18%). The relatively large proportion of long-distance links is explained by the flexible structure of the OCCM complex.

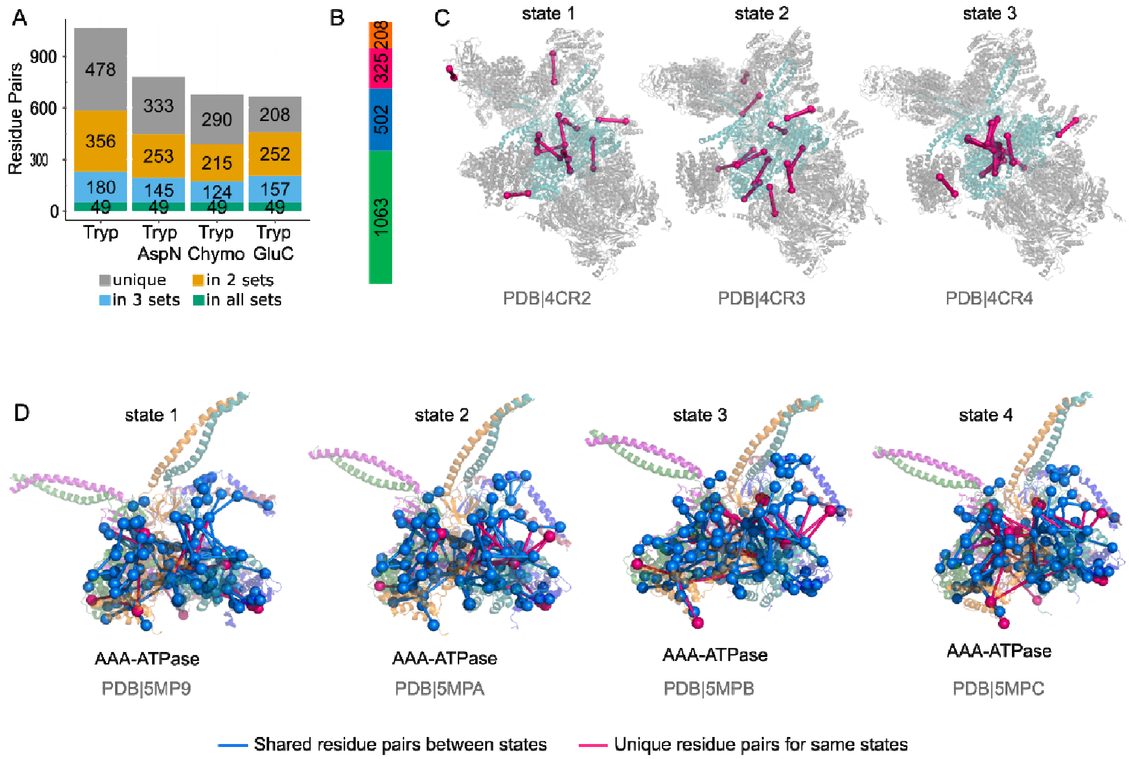

Appendix Figure S4: **The different states of the 26S Proteasome**

A. Overlap of the number of residue pairs for the four digestion conditions in the sequential digestion strategy. Sequential digestion complements trypsin digestion increasing the number of identified unique residue pairs.

B. Gain of unique residue pairs by using sequential digestion. The bar represents the number of unique residue pairs gained by adding AspN (blue), chymotrypsin (pink) and GluC (orange) to the digestion with trypsin (green).

C. Links matching the different states of the 26S proteasome described by Unverdorben *et al*. were mapped to the respective structures.

D. Links matching the different states of the AAA-ATPase dependent heterohexameric ring from the 26S proteasome described by Wehmer et al, 2017 were also mapped to the respective structures.

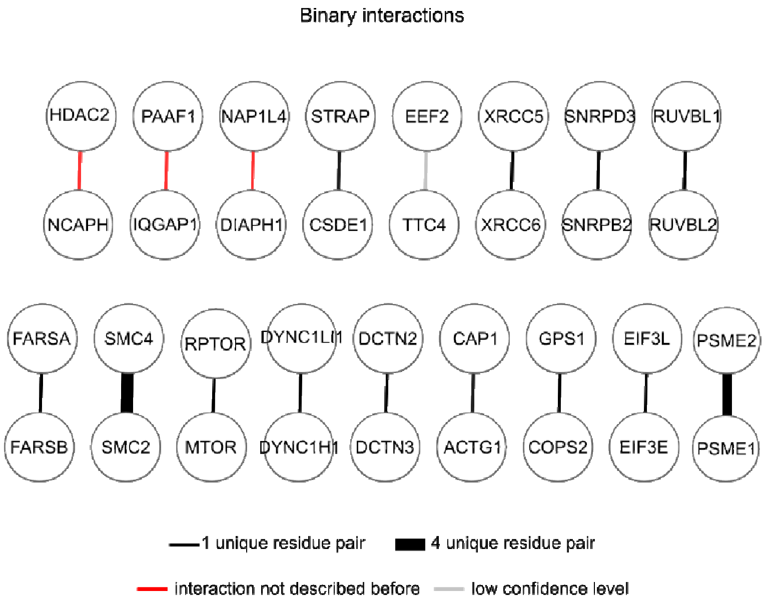

Appendix Figure S5:

Binary interactions for the cystosol of human cells

Apart from the identified networks, several binary interactions were also found in the cytosol of human cells represented in this figure.

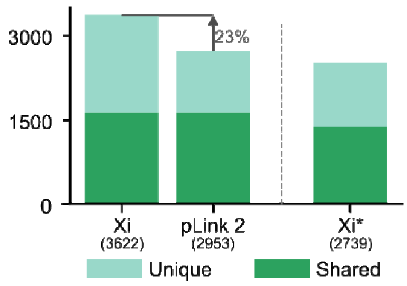

Appendix Figure S6: Comparison of the sequential digest protocol when combined with xiSEARCH or pLink 2

Search was done with BS3 specificity defined to be Lysine, Serine, Threonine and Tyrosine. Xi searches where run twice: once treating all linkable residues equal (as is done in pLink 2) and once (denoted Xi*) with giving priority to Lysine over Serine, Threonine and Tyrosine. A consequence of Xi* is that reporting a crosslink involving one of the side-reactions in favour of a near-by Lysine requires a substantial amount of evidence.
