# Supplementary material for: An integrated workflow for crosslinking mass spectrometry
Source: Mol Syst Biol. 2019 Sep 20;15(9):e8994. doi: 10.15252/msb.20198994 (PMC6753376; doi:10.15252/msb.20198994)
Supplement: Supplementary file 2 — Expanded View Figures PDF [file MSB-15-e8994-s002.pdf]

Expanded View Figures

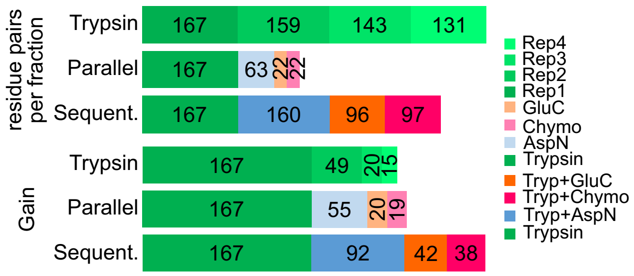

Figure EV1. Sequential digestion increases the number of identified unique residue pairs in a seven-protein mixture.
Links per fraction and gain for sequential digestion and the control experiments composed by an experiment using trypsin alone in four replicates and individual digestions with trypsin, AspN, chymotrypsin and GluC. Trypsin yields the higher number of links per sample followed by sequential digestion and individual digestions. However, sequential digestion yields the largest number of unique residue pairs when combining the data.

 © 2019 The Authors

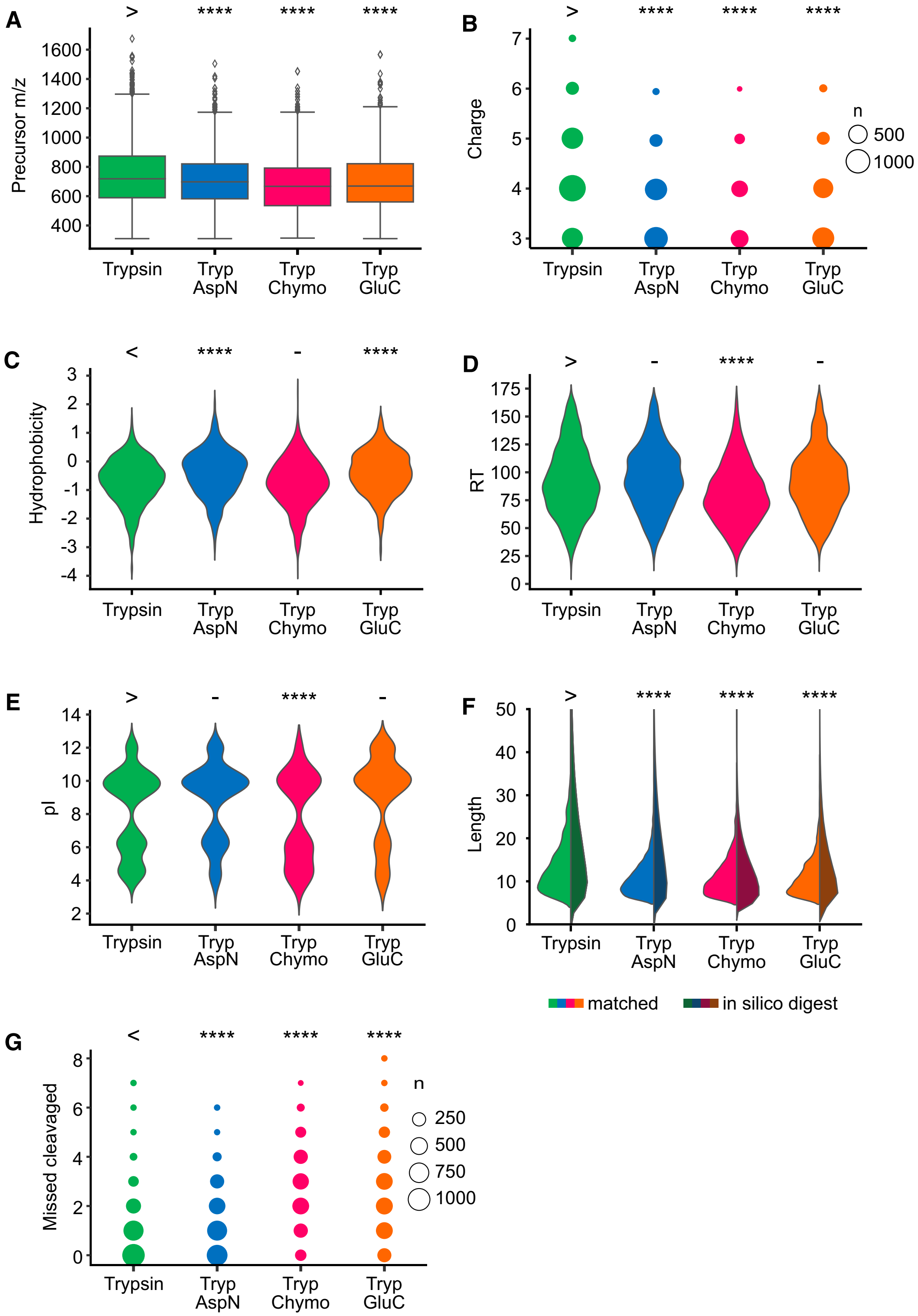
A
> **** **** ****
Precursor m/z
1600
1400
1200
1000
800
600
400
Trypsin
Tryp AspN
Tryp Chymo
Tryp GluC
B
> **** **** ****
Charge
7
6
5
4
3
n
500
1000
C
< **** - ****
Hydrophobicity
D
> - **** -
RT
E
> - **** -
pI
F
> **** **** ****
Length
matched
in silico digest
G
< **** **** ****
Missed cleavaged
n
250
500
750
1000

Figure EV2.

Figure EV2. Properties of crosslinked peptides (i.e. the two linked peptides are considered together) for a seven-protein mixture and each digestion condition.

A Precursor *m/z*. Sequentially digested peptides are smaller. Boxplot ranges represent the 25th (lower hinge) and 75th (upper hinge) percentiles, respectively. Middle line represents the median. Upper whisker and lower whisker are defined as follows: upper whisker = min(max(x), Q_3 + 1.5 * IQR), lower whisker = max(min(x), Q_1 − 1.5 * IQR) where IQR is the interquartile range (vertical size of the boxes).
B Observed charge state. Sequentially digested peptides have lower charge states.
C Calculated hydrophobicity
D Observed retention time (RT).
E Calculated pI.
F Peptide length, of both the observed peptides as part of a crosslink (left) and the number of unique crosslinkable peptides resulting from *in silico* digestion (right).
G Number of missed cleavages. Sequentially digested samples with trypsin + chymotrypsin and trypsin + GluC show more miss-cleavages than the other fractions.

Data information: For statistical testing, a one-sided Mann–Whitney *U*-test with continuity correction was used (Dataset EV4). All tests were carried out with trypsin as reference. The sign above the trypsin data (> or <) shows the direction of the alternative hypothesis. (****$P < 0.0001$, –: $P > 0.05$).

© 2019 The Authors

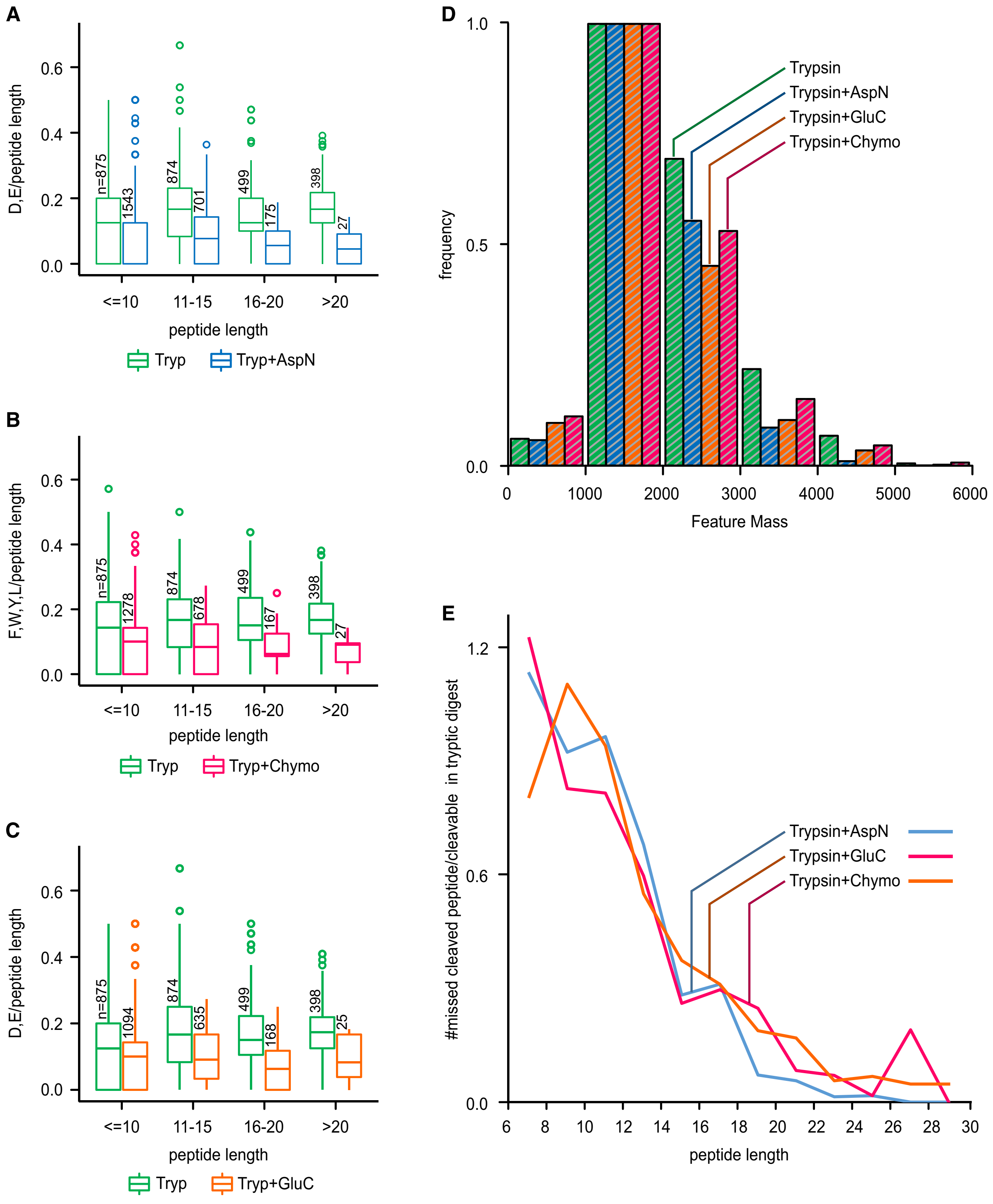
A
D,E/peptide length
peptide length
Tryp
Tryp+AspN
B
F,W,Y,L/peptide length
Tryp+Chymo
C
Tryp+GluC
D
frequency
Feature Mass
Trypsin
Trypsin+AspN
Trypsin+GluC
Trypsin+Chymo
E
#missed cleaved peptide/cleavable in tryptic digest

Figure EV3.

Figure EV3. Cleavage site protection in a BS^3-crosslinked 26S proteasome sample.

A–C We determined the number of available cleavage sites for each secondary enzyme (A: AspN, B: chymotrypsin, C: GluC) in both the trypsin (Tryp) dataset and their respective dataset (Tryp + AspN/Tryp + Chyno/Tryp + GluC). Boxplots show that the bigger the observed peptide is, the lower is its number of remaining cleavage sites, showing that large peptides with a higher number of cleavage sites were digested. In turn, smaller peptides contain a larger density of missed cleavage sites thereby indicating that short length protects peptides from digestion. Boxplot ranges represent the 25th (lower hinge) and 75th (upper hinge) percentiles, respectively. Middle line represents the median.

D Histogram of intensity weighted MS features as detected by MaxQuant for each digest. The sequential digests show a slight shift to lower masses, but most observed masses are between 1,000 and 2,000 Da.

E Protection of peptide from secondary cleavage measured as the number of missed-cleaved peptides in sequential digest divided by the number of peptides in the trypsin digest with potential cleavage sites for the second enzyme.

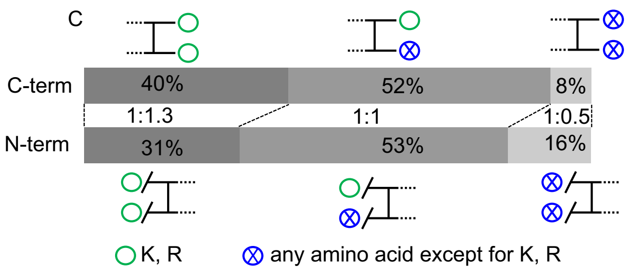

Figure EV4. Sequentially digested crosslinked peptides show a bias towards having C-termini that end in K or R.

In a seven-protein mixture, we see a bias towards tryptic C-terminal when compared to tryptic N-terminal showing that the increase of identification is in large part from the shorten but still tryptic looking peptides that are easier to identify by LC-MS/MS.

© 2019 The Authors

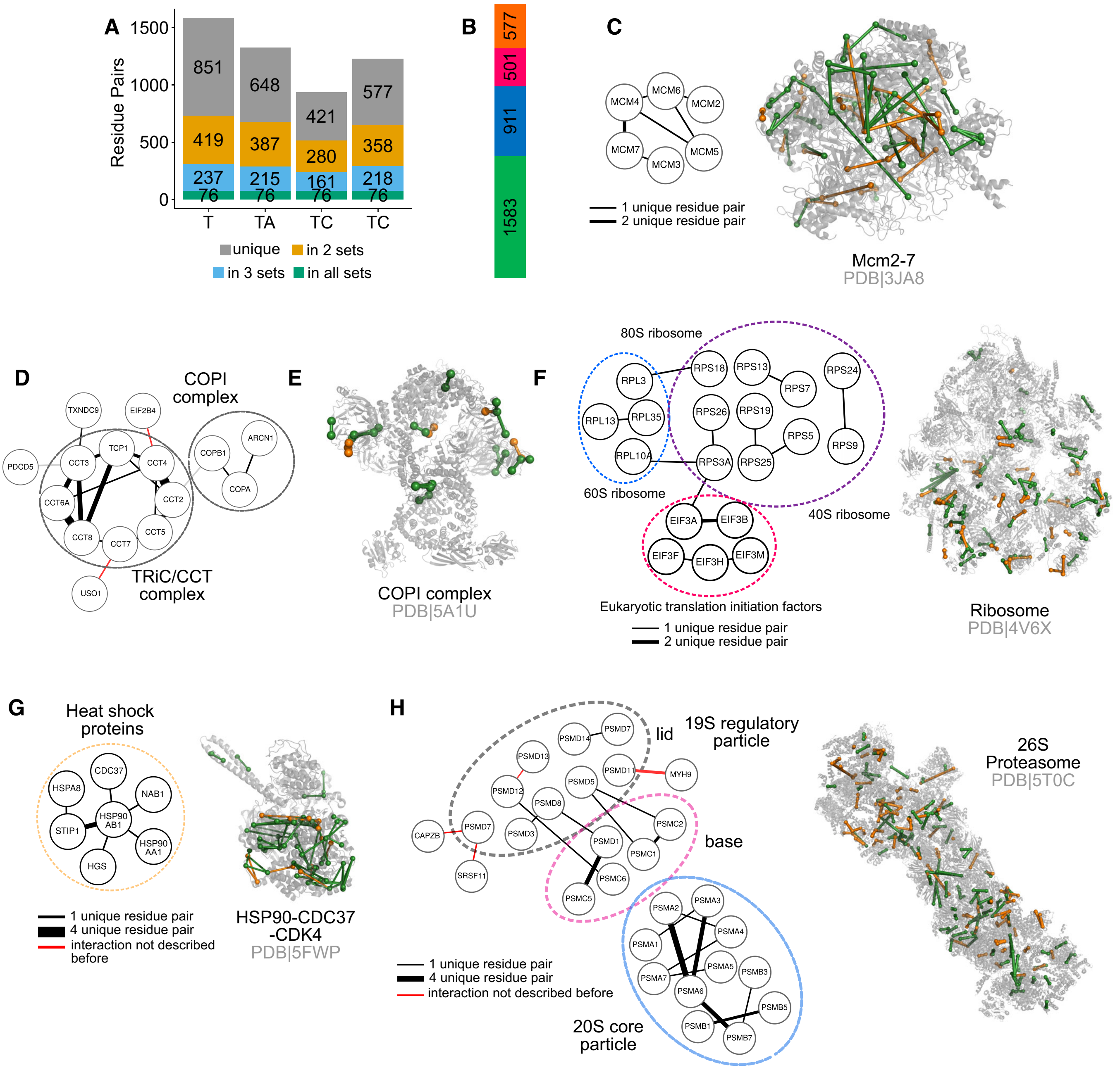

Figure EV5. CLMS of a human cytosol as a complex mixture using sequential digestion and xiSEARCH.
Tryptic links are displayed in green, and non-tryptic links are displayed in orange.

- A Overlap of the residue pairs obtained by the four digestion conditions applied. Sequential digestion complements trypsin digestion increasing the identified unique residue pairs.
- B Gain of unique residue pairs by using sequential digestion. The bar represents the number of unique residue pairs gained by adding AspN (blue), chymotrypsin (pink) and GluC (orange) to the digestion with trypsin (green).
- C The unique residue pairs identified for the MCM2-7 complex were used to generate a protein–protein interaction network and mapped into the crystal structure.
- D, E Protein–protein interaction network for the TRiC/CCT and COPI complexes was generated from the identified unique residue pairs. The unique residue pairs identified for the COPI complex were mapped into the respective crystal structure.
- F The unique residue pairs identified for the ribosome were used to generate a protein–protein interaction network and mapped into the crystal structure.
- G The unique residue pairs identified for the HSP90-CDC37-CDK4 complex were used to generate a protein–protein interaction network and mapped into the crystal structure.
- H The unique residue pairs identified for the 26S proteasome were used to generate a protein–protein interaction network and mapped into the crystal structure.

© 2019 The Authors

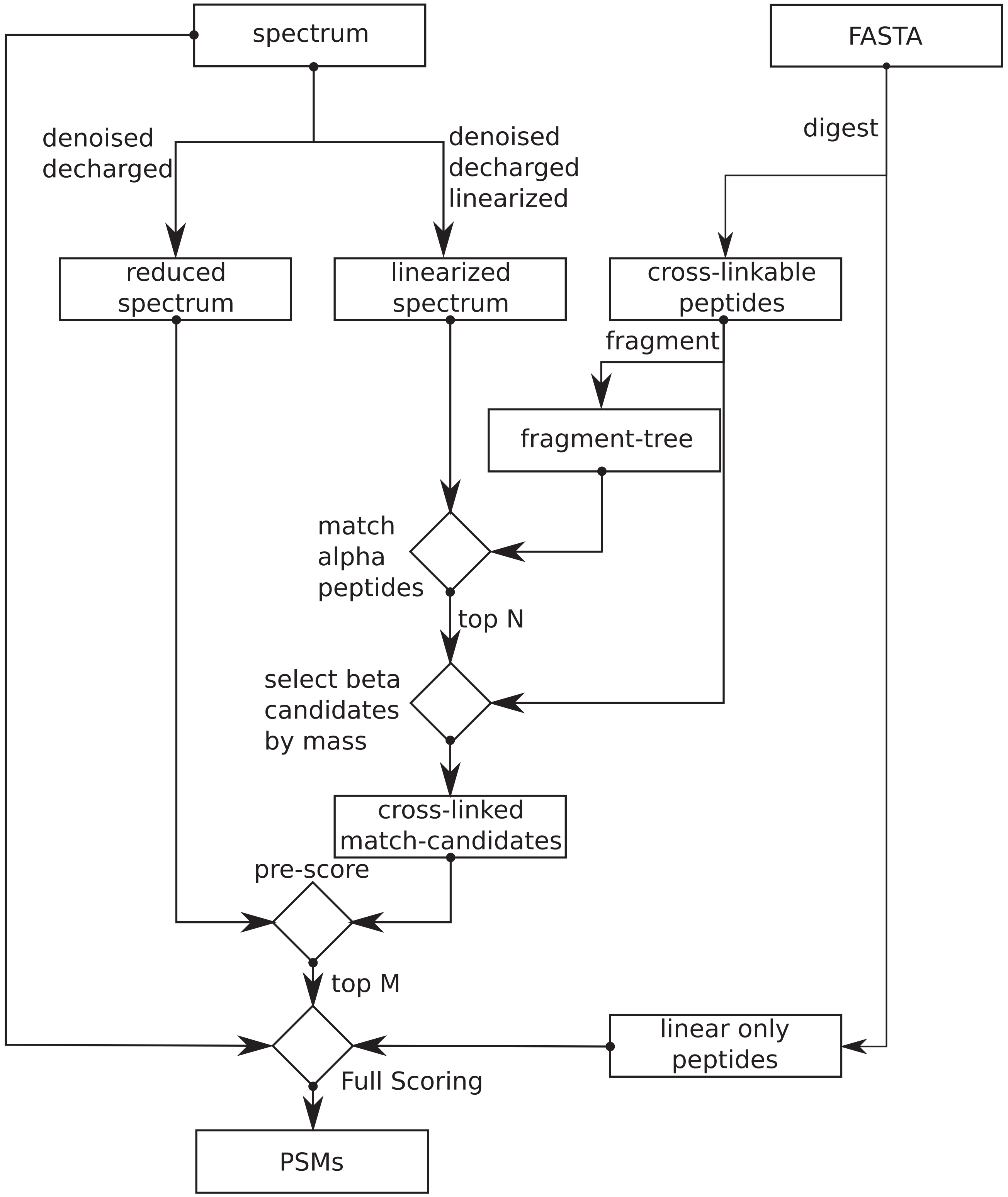

Figure EV6.

 © 2019 The Authors

Figure EV6. xiSEARCH follows a three-step approach.

In the first step, it tries to identify peptides with an unknown modification that explain the spectrum best. High-resolution data help in this step in two ways. Knowing the charge, and therefore the mass of a fragment, enables to predict whether a fragment is linear or crosslinked (Giese *et al*, 2016). This is important as only linear fragments are used to select peptide candidates. Second, with the knowledge that a fragment is probably crosslinked, we can invert the crosslinked fragments into their linear counterparts. By doing these two steps, we can de-convolute a crosslinked spectrum into a spectrum containing almost exclusively linear fragments of two independent" peptides. Additionally, the spectrum also gets de-charged and de-noised (linearised spectrum). To enable a fast identification, we create an in-memory representation of all primary fragment (e.g. b- and y-ions)-to-peptide relationships that can be derived from the search database (fragment tree). This fragment tree is then used to identify and quickly score alpha-peptide candidates in the linearised spectrum while ignoring the precursor mass. Secondly, the top-*n* candidates are then forwarded to the beta-peptide selection. Here, we take for each a peptide all peptides that fit the mass gap between the alpha-peptide candidate plus crosslinker and the precursor mass. The whole list of peptide-pair candidates is then again preliminarily scored. Third and finally, the top-m candidate pairs are then fully scored and reported.

© 2019 The Authors
